# Correspondence

**Published:** 1909-01

**Authors:** 


### Correspondence.

### Letter From "Proserpine"—Queen of Hades.

### Dr. Malchow's Book "The Sexual Life." A Criticism by "Proserpine."

A friend loaned me Dr. C. M. Malchow's book, *"The Sexual Life,"* to read. I turned page after page with increasing and almost horrified wonder and decreasing hope that ere I reached the end something would redeem the work from such judgment as I was forced to render against it. "Did my friend expect me to be enlightened, entertained, disgusted, or angered?" I asked myself. Summed up in one sentence, my judgment is: From first to last the writer howls a piteous howl over the fast crystallizing view that an evolving womanhood,—a womanhood rising into higher spheres where, emancipated from ignorance relative to her true place in race-life, she may subsist upon her own earnings of hand and brain instead of sacrificing upon the altars of sense—is swiftly widening the space between him and what, to him, are Heaven's very gates; i. e., lustful debauchery, either in or outside the wedded estate.

Nervous depression is one degree of neurotic ailment or disease. The *Century Dictionary and Encyclopedia* defines a neurotic woman, —not a neurotic *disease,* mind you,—thus: "A neurotic woman is sensitive, zealous, managing, self-forgetful; wearing herself for others." These are very fine and healthful qualities; and the *Dictionary* quotes as authority *"Buck's Handbook of Medical Science,"* p. 162. A neurotic disease or ailment, be it noted, is quite another matter from that of a neurotic woman. I distinguish carefully between the woman and the ailment or disease; and I do so specifically because since (unintentionally by myself) my letter to Dr. B. M. Worsham, of the State asylum for the insane at Austin, was published last May, he has stated in a written communication to me that: "Many cases of insanity result from neurotic elements or physical tendencies in either or both progenitors of the patient." The quotation may not be just his wording, but is essentially the same; and is still in my possession, but at this moment mislaid.

Just there, however, he granted my contention anent the large percentage of insanity as claimed in my first letter, being caused of maladjustment of, or excessive prodigality and waste, of sex forces. Take an instance of a neurotic woman who is well, and constituted according to the definition as quoted above from Buck. Burden her with maternity in about the degree supposed to be the duty of a milch cow—an animal with no other responsibility in life,—nightly and often daily sex demands during pregnancy as well as at any other time, the pinchings of poverty in a majority of cases, together with the innumerable trials of home-making during sickness and health. Then deny her the only real life-tonic she craves—love, pure and undefiled,—leave her starving heart unfed by simple, tender affection, while coldness and often cruelty are lavished freely, and a steady outpour of thoughtful attention to the husband's every need is expected, and what else save a neurotically diseased woman must be the logical outcome?

Just as this crisis Dr. Malchow suggests the horrible horror of applying local stimuli in order to excite a woman into a lustful condition. Near the bottom of page 38 we read: "One method (of producing sexual excitement) is by mental impressions, the other is by local stimulation or irritation." Concerning the last named method, page 303 records the following: "By the use of nux vomica, cannabis indica, resorcin, glycerine and a chlorinated compound of alcohol, a reaction to nervous depression may be obtained, and the woman put in the way of natural living." Horror or horrors! I could never have conceived of a more abominable theory, even among animals; and, too, for the self-evident purpose of merely gratifying his own animalism, proof of which assertion is again found on page 305: "To retain the (love) of a normal husband in the prime of life the wife should possess such qualifications as will supply *his* requirements." Insanity? The great wonder is that death doesn't mercifully precede insanity wherever and whenever such infamy is exercised. I know that if wives dared speak their true sentiments on this matter,—dared, lest they jeopardize their lot as to food and shelter,—ninety-nine out of every hundred would infinitely prefer the abnormal husband, if the normal one is cast after the mould Dr. Malchow is picturing. Did the learned author never see a wearied horse leap forward under the sting of the lash, and did he never note the deeper weariness that ever results from the "reaction" which must follow every unnatural action? And is the lash the true method of putting a horse in "the way of natural living?"

I had read thus far when I laid the book down and rang up a local druggist. "What is 'cannabis indica'?" I asked. "Indian hemp," he replied. "What are its effects?" I next inquired. "It produces delirium," he answered. And this, in part, is what Dr. Malchow advises in order to make a woman "live naturally." Merciful heaven! Whence cometh our help while this kind of "doctoring" is recognized as legitimate?

I once heard a pregnant definition of love, and believe no human effort could more completely define it, were volumes written in the attempt. It was: "Love *is* where *each* party to a wedded union supplies what the other needs." Can no one discover or invent some application for overbalanced centers of sensuality in men, causing *them* to live *naturally,* which, when applied will disperse the force to other centers where depletion abounds, and thus serve to light fires of heart and soul where they could radiate healing and balanced health instead of criminality, insanity and suicide? For this love definition names *needs,* not mere wants; a fact it may be worth noting; since "grownups," like children, often want what might kill them, while they *need* the wise action that enables them to live, and live free from neurotic ailments, too. In this brief sentence expounding the unexpoundable fact we have named love, the requirements are equally from, as to, the husband; a point Dr. Malchow seems to wholly disregard. Ought not a husband to possess the qualifications that will supply the *needs* of a wife in the prime of life—heart needs, though they may be? Is the normal wife so nearly imbecile as not to know what her real needs are that the normal husband must judge such a point for her, then proceed to create by "local irritation" a condition by which he can profit, regardless of either her pleasure or the welfare of herself and offspring? Let a woman speak who knows. The normal wife's needs are not generally of a sexual, but of a sexal nature; and the two differ widely, inasmuch as the latter are of the soul, the higher nature, and the former of the body or lower nature. Dr. Malchow dwells at length upon the necessity of developing the "special senses," meaning, doubtless, the sexual senses of the physical being. Has he never discerned that woman's strongest sensibilities are psychical, while man's are physical; and does he not appreciate the fact that it devolves ıully as much upon the man to develop his psychical as for woman to develop her physical senses in order to be to each other all that a true wedded union should be? This development of the psychical is done through pure love, and the cultivation of the object in which these psychic senses are to be evolved.

Illustrating: Suppose a man wishes to own a fruit garden; a place which, when cultivated, will support trees bearing figs, oranges, and numerous varieties of luscious provision. He selects ground deemed suitable, then places the plants in the earth where sunshine, dew, and all natural and needful elements will shower upon them. Need he doubt their productiveness? His only reasonable doubt could and would arise were he to neglect their proper cultivation. The simile is simple, yet profound. When a wife is loved there can be but one natural fruitage from her womanhood. Would the doctor scarify young trees he hoped would bear fruit and apply local stimuli therefor?

Woman weds with no specific thought of sexuality. She loves; and she thrills with happiness of a soul-kind. Her passions,—not lusts,—are pulsing with divine joy over the prospect of close association with her hero; and while this is sexal, it is not, I repeat, distinctly sexual. It only becomes sexual after the radiance of pure, unselfish love had been shed into her life for a few weeks or months, as the case may be. As is too often the rule, however, she speedily finds that her hero-husband knows nothing at all of soul-love, and finds no delight with her, aside from a constant descent to lusts of the flesh. *She* knows that passion, or sex-action on the psychic plane, transcends in exquisite ecstacies those on the physical, as the vibrations of the arc light transcend those of the tallow candle; yet, every effort she makes toward leading him to such knowledge only elicits the stunning declaration that she is abnormal; incomplete in some way, and ought to have recourse to local stimulus in order to arouse her latent sensuality! As if love,—life in activity,—would not do this very thing were he but to try applying *it* instead of seeking some material agency. Man's part toward woman is effected through love, the essence of divinity; and not by local application of drugs, or essences like cannabis indica. When woman as a wife once realizes that she retains no value in the estimation of her husband unless she is ever ready to sacrifice her womanhood upon the altar of lust, such realization is the death-blow to love in her heart; nor can she love with her body after the heart-fires have been extinguished. This condition I am striving to present is responsible for more domestic tragedy than all other causes combined, additional to the awful percentage of insanity resulting, where the sensitive wife prefers either the asylum or the grave to the humiliation attendant upon the divorce court.

The atmosphere resounds these days anent the fact of a declining motherhood; and herein lies the inner cause. Motherhood is woman's highest, holiest and most sacred aspiration; and nothing outside heaven can be likened to mother-love. The plane to which lust has degraded that holy function, however, has well-nigh destroyed its very fountain. The awful armies of criminal, refugee and insane were once tender babes. They lay upon a loving mother's breast; loved, always, though often unwanted, and their first faint movements heralding a mighty dread in the heart which sheltered them. Fear was bequeathed to the unborn through this dread, and from fear fruited sin and death,—woe, in prison and asylum and outside it; until the divine estate has come to be a tragedy far more terrible than that enacted on Calvary 2000 years ago. All this has resulted from an unrighteous use of righteous powers. On Calvary, the human body of one son of God (Good) was scourged and slain by the angered ignorance of a sin-blind people; and all because He sought to teach them a better, a more soul-saving knowledge of the Great Author of Being, and the laws He had given for their salvation. Today, the souls and bodies of countless thousands—men, women and children—are being sacrificed to a more dread ignorance,—ignorance of the science of life; and the scourge with which motherhood is being so smitten, and her little ones slain, is lust; murderous, destroying lust! And this is what Dr. Malchow is defending!

Is this speech too strong? Examine the heads of men and grown boys and an overwhelming average will be found to have abnormally developed lower-back brains, and, in many instances, almost a depression of the upper-back brain organ. Watch their private lives. Notice where they tend. The insane asylum and the penitentiary. That is sufficient comment. No speech can possibly be too strong wherewith to strike down the fortress of "uncleanness" that has been reared round about this enclosure (subject) wherein life is generated for such tragic ends.

"The holy estate of marriage" has been made an unholy estate of procreation whence issues a tide of sin-stamped humanity that is being driven forward to the grave; to death, here, and hereafter. It is a plea for these I am striving to sound forth where it may be heard. The poor unfortunates themselves are seldom heard from, nor their woes heeded by that other tide forming the glitter and the show-side of life, but the darker tide *is;* and it is by reason of the cause I am attacking. I don't know, as yet, by what methods I am to make my call in their behalf heard; but I am assured that sometime, somehow, the walls behind which rule the foes to purity inside the wedded estate will fall as fell the walls of Jericho before the commander of Israel.

In the centuries to come, forced or chanced motherhood will be regarded with sentiments of deeper horror than now thrill the sensitive reader who studies the dread doings of the Inquisition; and the race will wonder how it ever survived a period of its own evolution when devastating lust ruled in the homes of humanity.

Every man carries, in type, one of "Old Father Time's Scythes." Indeed, the destroying force of ungoverned procreative tides is the origin of that beautiful(?) picture. When woman accepts the dictum of any one as relates to its use, it mows her down like grass. Within the secret chamber where my own recreative agencies abide, I've seized the scythe, and recast it into a mighty stylus, with which I write letters I hope may be reflected from the very firmament. I refuse the cognition that any person possesses the right to dictate my duty to themselves. I am fully as competent to judge such a matter as any one; and I possess the right—not merely the privilege—of exercising that right, too.

Recently, some reverent admirer sent me a copy of *"The Marriage of Heaven and Hell,"* by William Blake. It is published by

John W. Luce & Company, of Boston. If the readers of the *Texas Medical Journal* wish, I will present them a review of that remarkable work. It contains one chapter of what the author calls "Proverbs of Hell." One reads: "Sooner murder an infant in its cradle than nurse unacted desires." I may add that I endeavored to ascertain whether the United States government permitted such matter to pass unmolested through the mails, and I have in my possession now a letter from the publishers, dated November 7, 1907, in which they say: "We will be pleased to send you one on receipt of price." I did not send for one, but the one I now have reached me through the mails, though I'm ignorant as to who paid me the wonderful compliment.

MRS. ELOISE SHEPHERD, B. O.
2806 Elgin Avenue, Houston, Texas.
Alias, *"Proserpine."*

Houston, Texas, December 15, 1908.

---

### International Medical Association.

SALTILLO, MEXICO, November 1, 1908.

*Dear Doctor:*

The next annual meeting of the International Medical Association of Mexico will be held in Tampico, January 20, 21, 22, and I am writing to urge you to attend and to kindly furnish the Association with a paper on some subject, of your own selection, pertaining to general medicine, to be read and discussed during the meeting.

Your paper (or its title) should be in the hands of the Secretary, Dr. J. S. Steele, Monterey, not later than January 1st, and should be as concise as justice to yourself and your subject will permit.

Many interesting papers have been promised, many members have advised the Secretary of their intention of attending, besides the assurances from prominent members of the profession from the United States that they will attend assures us of a large, an enthusiastic, a pleasant and a profitable meeting, and we indulge the hope that you will also be present with an article commensurate with your recognized ability and fluency.

Yours fraternally,
R. H. L. BIBB,
Chairman Sec. Genl. Medicine.

### Song of the Shirt[waist].

Oh, fickle fashion, hasten to revoke that mad decree
You issued to your votaries two years ago, or three,
When all the women of the land their shirtwaists did sidetrack,
To get the one you favored most—that
Buttoned
Down
The
Back!

If hubby's gone and servant girl has afternoon away,
And children, too, are off at school, or out perhaps at play,
You're due at Club, Bridge Whist or Tea, there's none alas, alack!
To fasten up the waist for you that
Buttons
Down
The
Back!

And so perforce the sex performs contortions day by day,
And acrobatic feats and dextrous moves of vertebrae,
Make heroes every day of those who've learned the ready knack
Of fastening up the waists that
Button
Down
The
Back!

—*Thekla M. Brumby in Texas Health Bulletin.*

### Sick!

When mother's sick the house is all
So strangely hushed in room and hall!
But mother never will admit
She's suffering a single bit!
She won't let people do a thing—
There's nothing anyone can bring—
She just lies there and tries to fix
Herself by cunning little tricks!
And as for the doctor—why the word
She scouts as being most absurd.

And when he comes he has to guess
At symptoms that she won't confess;
And then he's apt to frown and say:
"You should have had me right away.
I'll come again this evening"—for
It's bed, you see, a week or more!

When father's sick—I tell you, now,
You ought to hear the dreadful row!
The talk of "dying" and the groans!
The orders in convulsive tones!
The hasty runnings to and fro
To rearrange the pillow—so;
To fix hot-water bag and shade;
For mustard plaster, lemonade!
Appeals to get the doctor, quick—
And "Can't you see I'm awful sick?"
And then the doctor sits and hears
While father grunts his pains and fears.
He leaves some drops, and tells us: "Hum!
Unless I'm needed I shan't come
Again. I think he'll do all right."
And father's up, perhaps, by night!

—*Edwin L. Sabin, in Century.*

---

THE TREATMENT OF CONVULSIONS (Due to Teething, Gastro-Intestinal Causes, etc.) in a Child of Three Years.—

I.

*Purgative Enema:*

℞ Senna leaves .................... 5.0 grammes
Sodium sulphate ................ 10.0 grammes
Boiling water ....................150.0 grammes

F. S. A. To be given warm.

*Internal Purge:*

℞ Calomel ..........................0.20 gramme
Lactose ..........................2 gammes

One powder in a little sweetened water.

II.

Milk diet. Quiet; a darkened room, and, to relieve tendency to spasmodic contractions:

℞ Antipyrine ......................0.30 gramme
Cacao butter ......................2.0 grammes

F. S. A. One suppository. Use one daily.

Or,

| | | |
|---|---|---|
| ℞ Potassium bromide, | | |
| Sodium bromide, | aa | 1 gramme |
| Strontium bromide, | | |
| Syrup of orange peel | | 20.0 grammes |
| Linden flower water | | 80.0 grammes |

Four teaspoonfuls daily. (Increase as needed.)

III.

Warm baths at 36° C., daily or twice daily, lasting from fifteen to twenty minutes each, and, in case of violent convulsions, ice bags to the head, mustard plasters to the feet, and inhalations of ether or chloroform.—ALFRED MARTINET (*Presse medicale*).

---

HYPERACUTE GONORRHEA.—

I.

| | | |
|---|---|---|
| ℞ Sod. bicarb., | aa | ʒss |
| Sod. bromid, | | |
| Tr. belladonnæ | | ʒi |
| Aq. menth, pip., | aa | ℥iii |
| Syr. acaciæ, | | |

Sig.: ℥ss every three hours.

II.

| | |
|---|---|
| ℞ Potass. bicarb. | ℥i |
| Tr. hyoscyami, | ʒss |
| Ext. kavæ kavæ, fl., | |
| Aquæ, ad | ℥viii |

A tablespoonful in a wineglassful of water three times daily.

---

PAINLESS TOOTH EXTRACTION.—A correspondent of the *American Journal of Dental Science* says: The following is my solution for the painless extraction of teeth:

| | | |
|---|---|---|
| ℞ Cocaine hydrochloride, | | |
| Carbolic acid (cryst), | aa | gr. x |
| Gum camphor, | | |
| Chloral hydrate, | | |
| Aq. dest., q. s. ad | | ℥iv |

Rub the camphor and chloral together until liquefied; add acid, cocaine, and lastly water, and filter. Sig. For painless extraction I have had it compounded a great many times at 25 cents for four ounces. Compare the price with your dollar-an-ounce solution, and compare it in any other way, for it will bear comparison. I compound my own solution, and thus vouch for its accuracy. It takes effect immediately, and I have my first case to hear from where sloughing followed its administration.—*Albright's Office Practitioner.*

